# Three-dimensional artificial intelligence-based computed tomography analysis of lower limb muscle volume and fatty degeneration in varus and valgus knee osteoarthritis: a single-center retrospective study

**DOI:** 10.1186/s12891-026-10034-5

**Published:** 2026-06-05

**Authors:** Yoshinobu Hyakuda, Takuma Miyamoto, Munehiro Ogawa, Akihito Kawai, Hiroto Nishimura, Ryota Hara, Yusuke Inagaki, Keisuke Uemura, Masaki Takao, Kohei Kono, Mazen Soufi, Yoshito Otake, Kenji Kawamura

**Affiliations:** 1 Department of Orthopaedic Surgery, Nara Medical University, 840 Shijo-cho, Kashihara, Nara 634-8521 Japan; 2 Department of Sports Medicine, Nara Medical University, Kashihara, Japan; 3 The Center for Rheumatic Diseases, Nara Medical University, Kashihara, Japan; 4 Department of Rehabilitation Medicine, Nara Medical University, Kashihara, Japan; 5 Department of Orthopaedic Medical Engineering, Osaka University Graduate School of Medicine, Suita, Japan; 6 Department of Orthopaedic Surgery, Ehime University Graduate School of Medicine, Toon, Japan; 7 Program of Information and Communication Engineering, Faculty of Engineering, University of Miyazaki, Miyazaki, Japan; 8 Division of Information Science, Nara Institute of Science and Technology, Ikoma, Japan

**Keywords:** Knee osteoarthritis, Varus deformity, Valgus deformity, Muscle volume, Fatty degeneration, AI-based segmentation, Three-dimensional CT

## Abstract

**Background:**

Despite the high prevalence of knee osteoarthritis, the anatomical factors that characterize distinct deformity patterns remain unclear. This study aimed to compare the three-dimensional (3D) lower-limb musculature between varus and valgus knee osteoarthritis (OA) using an artificial intelligence (AI)-based computed tomography (CT) segmentation, evaluating whole-muscle volume, fatty degeneration, and morphological differences to inform targeted prevention, treatment, and rehabilitation strategies.

**Methods:**

This retrospective study included 75 patients who underwent CT imaging before total knee arthroplasty for end-stage knee OA. Patients were categorized into valgus (femorotibial angle [FTA] ≤ 176°) and varus (FTA > 176°) groups. A validated Bayesian U-Net-based AI segmentation model was used to reconstruct 3D musculature and quantify muscle volume and Hounsfield unit (HU) values. Twenty-three muscle compartments were analyzed. For the vastus medialis and tensor fasciae latae (TFL), intramuscular fat volume was additionally quantified using Mimics to calculate fat percentage. Between-group comparisons were performed using the Mann–Whitney U test, and false discovery rate correction was applied for multiple comparisons.

**Results:**

The valgus group (*n* = 16) demonstrated significantly smaller muscle volumes in the biceps femoris, TFL, and superficial posterior compartment than the varus group (*n* = 59) after false discovery rate correction. The HU value of the TFL was significantly lower in the valgus group. The vastus medialis showed a lower HU value before correction; however, this difference did not remain significant after false discovery rate correction. Mimics-based fat quantification revealed no significant difference in the fat percentage of the vastus medialis, whereas the TFL showed significantly higher fat infiltration in valgus knees. Correlation analyses using FTA as a continuous variable showed that greater valgus alignment was associated with lower TFL muscle volume, lower TFL HU values, and greater TFL fat infiltration.

**Conclusions:**

AI-assisted CT-based 3D analysis revealed distinct muscle morphologies and fatty degeneration patterns in varus and valgus knees of patients with OA. Valgus knees exhibited characteristic reductions in the posterior and lateral muscle groups and increased fatty infiltration of the TFL. These deformity-specific muscular alterations may inform personalized rehabilitation strategies and help elucidate the factors contributing to the progression of OA.

**Trial registration:**

Not applicable.

**Supplementary Information:**

The online version contains supplementary material available at https://doi.org/10.1186/s12891-026-10034-5.

## Background

Knee osteoarthritis (OA) remains one of the leading causes of knee pain and long-term disability worldwide [1], and is also the most common form of arthritis [2]. Exploring the differences in the musculature around the knee between varus and valgus knees in osteoarthritis can help understand knee joint function, and thereby help prevent disability and establish effective rehabilitation strategies [3]. Orthopedic surgeons employ various approaches to correct deformities, particularly during total knee arthroplasty (TKA) or osteotomy [4]. However, the factors that promote the progression of varus or valgus deformities remain poorly understood. To enhance surgical planning and deepen our knowledge of the structural factors associated with specific OA progression patterns, the anatomical differences between varus and valgus knees must be investigated.

While previous studies have focused primarily on quadriceps strength and periarticular muscle function, recent investigations have examined anatomical variations, neuromuscular control, and biomechanical effects associated with misalignment [5, 6]. Several reports have described muscle volume and fatty degeneration in knee OA, with most studies relying on single-slice computed tomography (CT) or magnetic resonance imaging (MRI) [3, 7]. Since the three-dimensional (3D) architecture of muscles varies, single-slice assessments cannot accurately reflect muscle volume or morphology [8]. Fatty degeneration is also known to differ across slices within the same muscle, further limiting the accuracy of single-slice evaluations [9, 10].

A 3D assessment is generally preferable; however, manual segmentation across multiple slices is labor-intensive and impractical when the analysis of many muscles is required. Recent studies have applied artificial intelligence (AI)-based muscle segmentation to CT and MRI scans to address this issue [10, 11].

Using CT-based 3D analysis with an AI segmentation model, the present study aimed to comprehensively evaluate muscle length, volume, fatty degeneration, and morphology across the entire lower limb musculature and to identify differences between varus and valgus knees in knee OA. These findings may help improve the prevention, treatment, and rehabilitation of this patient population. Based on biomechanical considerations, we hypothesized that varus knees develop a relatively stronger lateral musculature, such as the tensor fasciae latae (TFL), whereas valgus knees develop a relatively stronger medial musculature, such as the vastus medialis.

## Methods

### Study design

**Fig. 1:**
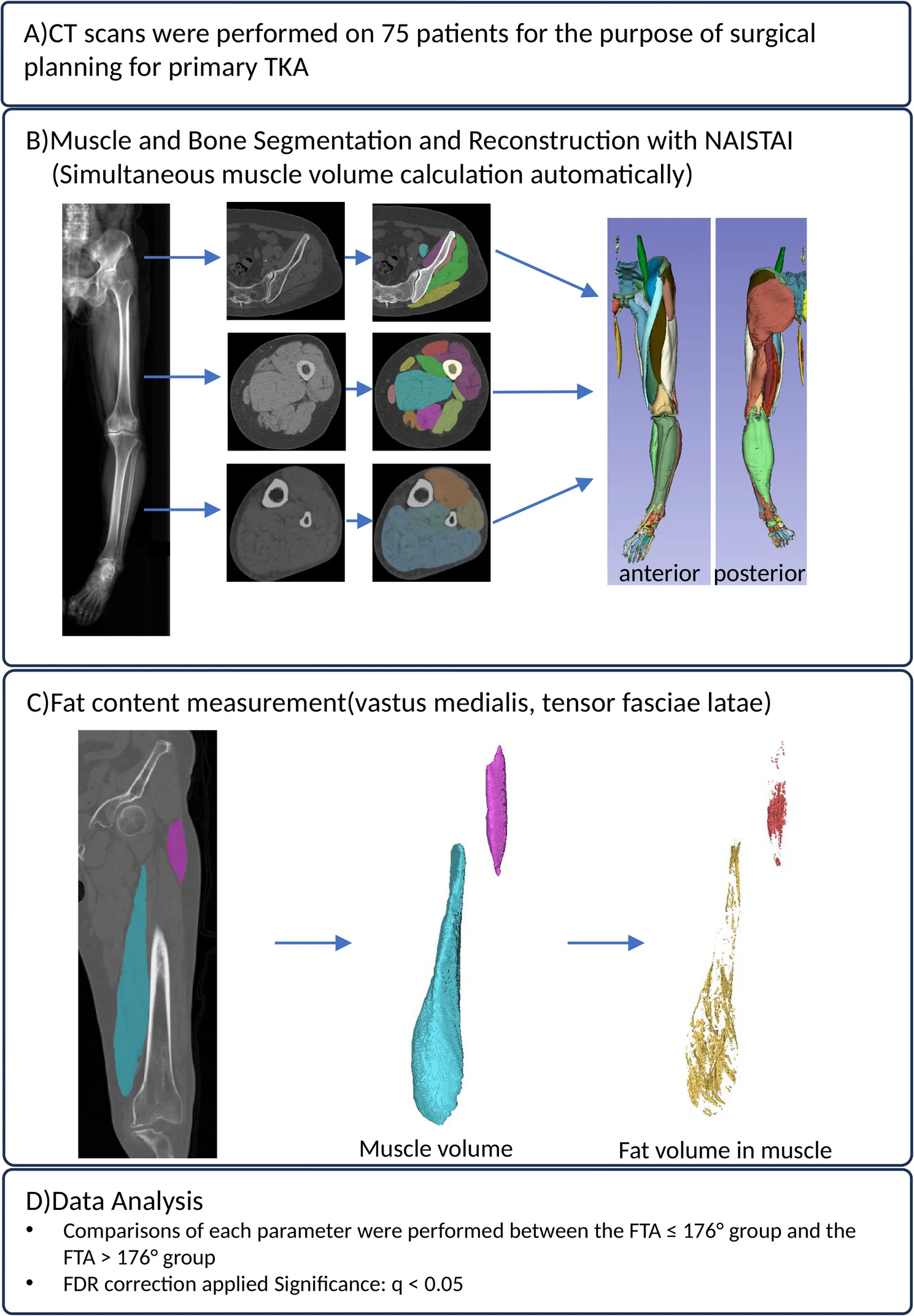
Workflow of AI-based 3D CT muscle analysis in varus and valgus knee osteoarthritis. This figure illustrates the overall study workflow. **A **Preoperative CT scans were obtained from the hip to the foot for 75 patients undergoing primary TKA. **B** Three-dimensional reconstruction and segmentation of muscles and bones were performed automatically using the AI deep learning model, enabling the simultaneous calculation of muscle volume and mean HU values. **C** Additional Mimics-based segmentation was performed for the vastus medialis and tensor fasciae latae to quantify intramuscular fat volume and calculate fat percentage.** D** Statistical comparisons were conducted between the valgus group (FTA ≤ 176°) and the varus group (FTA > 176°). False discovery rate correction was applied to analyses of the 23 muscles or muscle compartments.AI, artificial intelligence; 3D, three dimensional; CT, computed tomography; TKA, total knee arthroplasty; HU, Hounsfield unit; FTA, femorotibial angle

This retrospective observational study was approved by the Institutional Review Board of Nara Medical University (Approval No. 4117). Because this retrospective study used anonymized clinical data, the requirement for written informed consent was waived, and an opt-out document was publicly posted on the hospital website in accordance with the institutional policy. This study was conducted in accordance with the principles of the Declaration of Helsinki. The study methodology is illustrated in Fig. 1.

### Data collection

We reviewed 106 consecutive patients who underwent unilateral TKA for knee OA at Nara Medical University Hospital between January 1, 2024, and May 31, 2025. Fifteen patients lacked preoperative CT scans from the hip to the foot, and 16 patients with a history of ipsilateral lower-limb surgery were excluded. None of the patients had any missing data for muscle volume or Hounsfield units (HU). Finally, 75 patients were included in the analysis (mean age, 73.6 ± 7.7 years; mean height, 155.1 ± 8.2 cm; mean weight, 63.8 ± 12.0 kg; mean body mass index [BMI], 26.4 ± 4.1 kg/m^2^). The cohort comprised 23 men and 52 women.

**Fig. 2:**
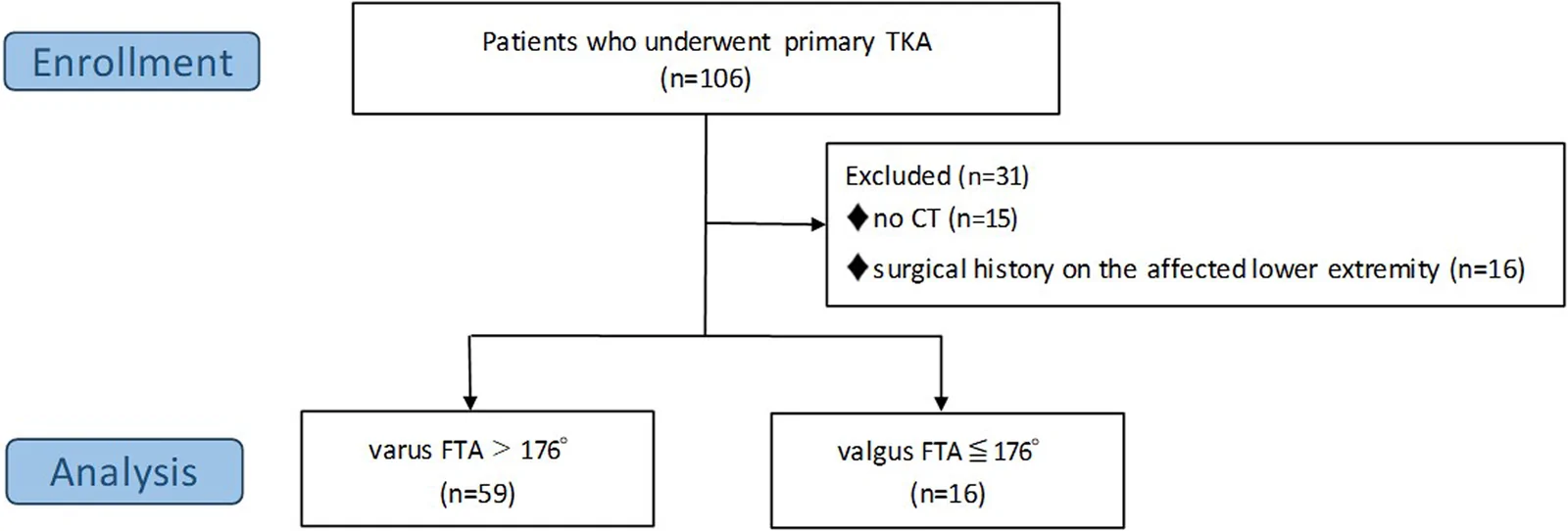
Flowchart of Patient Inclusion and Group Allocation. Among 106 consecutive patients scheduled for primary TKA, 15 were excluded because of incomplete computed tomography (CT) imaging and 16 because of previous ipsilateral lower limb surgery. A total of 75 patients were included and categorized into two groups: valgus knees (FTA ≤ 176°, *n* = 16) and varus knees (FTA > 176°, *n* = 59). TKA, total knee arthroplasty; FTA, femorotibial angle

Patients with a femorotibial angle (FTA) ≤ 176° were categorized into the valgus group, whereas those with an FTA > 176° were categorized into the varus group (Fig. 2).

FTA was measured on weight-bearing full-length lower-limb radiographs following a predefined and standardized measurement protocol using established anatomical landmarks. The threshold of 176° for categorization of varus alignment was selected based on prior reports of normative femorotibial angle values, with average FTAs of approximately 176° in women and 178° in men in the general adult population [12]. The former value was selected as the study population consisted predominantly of female patients. CT imaging was performed using a SOMATOM Definition AS (Siemens AG, Munich, Germany) with the following parameters: tube voltage, 120 kV; tube current, 160 mA; slice thickness, 1.0 mm; and matrix size, 512 × 512. The scanning range extended from the hip to the foot. At our institution, full-length lower-limb CT scans spanning from the hip to the foot are routinely obtained for preoperative 3D planning of primary TKA using ZedKnee (LEXI Co., Ltd., Tokyo, Japan). This imaging protocol serves to evaluate the lower-limb mechanical axis and guide component alignment decisions within the surgical planning workflow. Accordingly, the CT scans analyzed in this study were obtained for routine clinical purposes and were not performed specifically for the purpose of the present retrospective study. Clinical and imaging data were extracted from the institutional picture archiving and communication system and the electronic medical record system by a study investigator, covering the period from June 2025 to September 2025.

AI-based segmentation was used to reconstruct the 3D musculature of the lower limb and compute the muscle volume and HU values, used as imaging-based surrogates of fatty degeneration. We used a deep learning model based on a fully convolutional neural network with a Bayesian U-Net architecture [13, 14], which was previously shown to provide high reliability for the muscle segmentation of bones and soft tissues from hip-to-knee CT [11]. The segmentation accuracy of this AI model demonstrated a Dice coefficient of 0.958 ± 0.020, a mean surface error of 0.560 ± 0.770 mm, and measurement accuracy for muscle volume and mean HU of 2.690 ± 2.870% and 0.647 ± 0.707 HU, respectively [10]. The Bayesian U-Net model applied in this study was originally validated on hip-to-knee CT images; consequently, its application to the full hip-to-foot field of view, including the lower-leg compartments, may introduce a domain shift related to the distal leg anatomy, field-of-view truncation, or reconstruction settings, which may affect the segmentation accuracy and downstream HU and muscle volume estimates. All segmentation processes underwent visual quality control by an orthopedic surgeon, which identified no segmentation errors requiring correction or exclusion. The AI-based segmentation framework was developed in collaboration with the Nara Institute of Science and Technology. Although the model had been previously quantitatively validated for hip-to-knee CT images, formal quantitative validation specifically for the lower-leg compartments was not undertaken in the present study.

Twenty-three muscles or muscle compartments were analyzed: adductor group, biceps femoris, gluteus maximus/medius/minimus, gracilis, iliacus, obturator externus/internus, pectineus, piriformis, psoas major, rectus femoris, sartorius, semimembranosus, semitendinosus, TFL, and vastus lateralis/intermedius, vastus medialis; and the anterior, lateral, deep posterior, and superficial posterior compartments of the lower leg. These muscles and compartments were selected based on their biomechanical relevance to lower-limb alignment and the feasibility of consistent segmentation.

For the vastus medialis and TFL, additional segmentation was conducted using Materialise Mimics version 26 (Materialise, Leuven, Belgium) to quantify muscle volume and intramuscular fat volume. The fat percentage was calculated using the following formula:

$$\:Fat\:percentage\:\left(\%\right)=\:\frac{Fat\:volume}{Muscle\:volume}\times\:100$$

Fat CT attenuation was referenced to buttock fat in each case, and voxels within the muscle-matched fat attenuation range were segmented as intramuscular fat [15].

### Statistical analysis

Normality was assessed using the Shapiro–Wilk test. Comparisons of age, height, weight, and BMI were conducted using the Student’s *t*-test, while sex was compared using the chi-square test. Muscle volume and CT attenuation values were not normally distributed; therefore, comparisons between the varus and valgus groups were conducted using the Mann–Whitney *U* test. Statistical significance was set at *p* < 0.05. Analyses were performed using SPSS version 28.0 (IBM Corp., Armonk, NY, USA). Given the large number of parallel comparisons across 23 muscles/compartments and the multiple outcome metrics, the false discovery rate was controlled using the Benjamini–Hochberg procedure to reduce the risk of type I errors, while maintaining adequate sensitivity. Furthermore, Spearman’s rank correlation analysis was performed to evaluate the relationships between FTA as a continuous variable and representative muscle parameters, including TFL muscle volume, TFL HU value, TFL fat infiltration, biceps femoris muscle volume, and superficial posterior compartment volume. Scatter plots were subsequently generated to visualize these correlations between FTA and each of these muscle parameters.

## Results

The valgus and varus groups included 16 and 59 patients, respectively. The baseline characteristics did not differ significantly between the groups (Table 1).

**Table 1:** Baseline Characteristics of the Study Population

	Case (*n*)	Age	Sex	Height (cm)	Weight (kg)	BMI (kg/m^2^)
All	75	73.6 ± 7.7	F:52 M:23	155.1 ± 8.2	63.8 ± 12.0	26.4 ± 4.1
Varus	59	73.1 ± 7.4	F:42 M:17	155.1 ± 8.0	64.0 ± 11.6	26.5 ± 4.0
Valgus	16	75.1 ± 8.8	F:10 M:6	155.1 ± 9.0	62.9 ± 13.8	26.0 ± 4.6
*p*-value (α = 0.05)		0.360	0.504	0.977	0.735	0.632

Baseline demographics including age, height, weight, and BMI are presented for the valgus and varus groups. No statistically significant differences were observed between groups

**Fig. 3:**
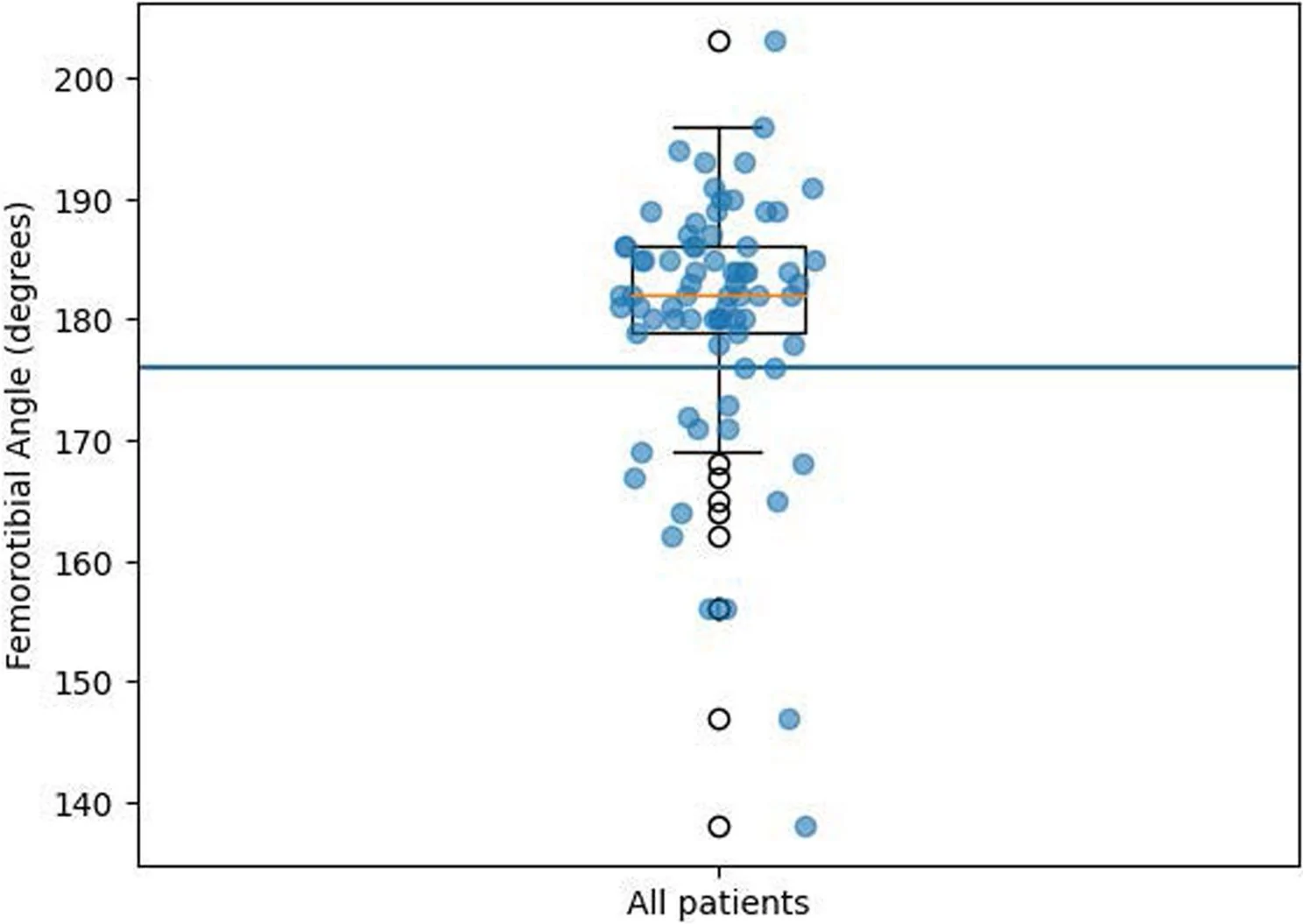
Distribution of femorotibial angle (FTA) values. The box plot shows the median and interquartile range, with individual data points overlaid. The horizontal line indicates the predefined threshold of 176° used for group classification

We reported the distribution of FTA values in each group, summarized as the median [IQR] and range. Overall, the median FTA was 182° [179–186], with a range of 138–203°. Only a small number of cases were located near the prespecified threshold of 176°, indicating a limited overlap between groups around the cutoff (Fig. 3).

After false discovery rate correction, muscle volumes were significantly smaller in the valgus group in the biceps femoris, TFL, and superficial posterior compartment than in the varus group (Table 2).

**Table 2:** Comparison of Muscle Volume Between Varus and Valgus Knees

Muscle/Compartment	Varus (vol) [cm^3^] (median [IQR])	Valgus (vol) [cm^3^] (median [IQR])	Between-group difference (median difference [95% CI])	*p*	q
adductor	611.8 [508.0–714.6]	494.4 [393.3–679.2]	96.4 (− 6.2 to 194.2)	0.660	0.759
biceps femoris	203.9 [165.3–256.9]	158.8 [112.3–185.8]	49.9 (19.1 to 89.2)	0.003	0.023
gluteus maximus	677.3 [586.1–762.1]	590.4 [496.5–764.6]	64.9 (− 50.1 to 155.1)	0.289	0.350
gluteus medius	256.2 [220.6–300.5]	252.1 [200.9–294.7]	2.0 (− 29.4 to 40.3)	0.877	0.877
gluteus minimus	55.1 [47.9–60.1]	48.1 [42.6–56.2]	4.9 (− 2.0 to 11.2)	0.162	0.274
gracilis	50.9 [42.3–69.1]	48.2 [41.3–65.7]	1.7 (− 9.2 to 13.2)	0.776	0.811
iliacus	90.9 [80.6–115.6]	83.1 [67.3–106.6]	8.8 (− 6.9 to 22.9)	0.274	0.350
obturator externus	36.3 [31.2–41.7]	31.2 [28.5–35.3]	4.0 (− 0.7 to 8.4)	0.085	0.244
obturator internus	36.0 [31.2–40.4]	37.4 [27.5–41.8]	0.6 (− 4.6 to 5.8)	0.776	0.811
pectineus	32.0 [24.9–38.3]	27.8 [23.6–32.9]	3.3 (− 1.5 to 8.5)	0.179	0.274
piriformis	19.7 [16.1–22.9]	21.7 [18.1–25.5]	−2.3 (− 5.3 to 0.9)	0.140	0.274
psoas major	77.5 [61.5–93.8]	59.1 [43.9–96.0]	12.6 (− 6.6 to 29.1)	0.155	0.274
rectus femoris	121.5 [93.2–144.7]	100.8 [76.1–121.9]	17.2 (− 0.5 to 35.7)	0.057	0.244
sartorius	98.4 [76.7–120.9]	79.9 [63.1–104.0]	14.9 (− 2.0 to 32.7)	0.081	0.244
semimembranosus	148.4 [122.9–177.1]	133.2 [108.3–162.2]	15.7 (− 9.4 to 42.2)	0.224	0.322
semitendinosus	120.3 [96.1–139.6]	97.4 [78.5–121.8]	18.1 (0.3 to 36.0)	0.048	0.244
tensor fasciae latae	52.2 [41.2–65.8]	34.5 [24.8–43.9]	18.2 (8.5 to 28.7)	< 0.001	< 0.001
vastus lateralis intermedius	518.8 [411.8–609.7]	432.8 [326.4–594.5]	73.0 (− 29.0 to 162.4)	0.179	0.274
vastus medialis	233.1 [183.6–266.6]	194.7 [148.5–223.0]	31.1 (− 8.8 to 68.5)	0.124	0.274
anterior compartment	149.0 [108.6–185.3]	129.1 [112.1–156.5]	15.3 (− 14.7 to 40.0)	0.239	0.323
lateral compartment	69.7 [58.7–91.3]	59.0 [41.5–74.1]	11.8 (− 1.1 to 27.2)	0.081	0.244
deep posterior compartment	188.0 [156.7–223.4]	169.5 [154.7–189.8]	19.1 (− 5.1 to 46.3)	0.121	0.274
superficial posterior compartment	496.8 [425.8–567.9]	354.7 [235.6–437.1]	149.3 (74.5 to 227.7)	0.001	0.012

The muscle volume of 23 muscles or muscle compartments was compared between groups. Significant reductions in the valgus group after false discovery rate correction were observed in the biceps femoris, tensor fasciae latae, and superficial posterior compartment. Values are presented as median [interquartile range]. Between-group differences are presented as Hodges–Lehmann median differences with 95% confidence intervals (CIs). Mann–Whitney U test was used for between-group comparisons. *p* values are reported as unadjusted values, and q values represent false discovery rate–adjusted p values calculated using the Benjamini–Hochberg procedure. Statistical significance was determined based on q < 0.05. IQR, interquartile range

In the supplementary analysis using body-weight-normalized muscle volume, the major findings were generally consistent with those of the primary analysis. The valgus group showed significantly lower normalized muscle volumes in the biceps femoris, TFL, and superficial posterior compartment after false discovery rate correction (Supplementary Table 1). 

After false discovery rate correction, the mean HU value was significantly lower in the valgus group exclusively for the TFL (Table 3). The vastus medialis showed a lower HU value before correction; however, this difference did not remain statistically significant following false discovery rate correction.

**Table 3:** Comparison of Mean Hounsfield Unit (HU) Values Between Varus and Valgus Knees

Muscle/Compartment	Varus (HU) (median [IQR])	Valgus (HU) (median [IQR])	Between-group difference (median difference [95% CI])	*p*	q
adductor	38.9 [34.2–42.4]	34.6 [30.4–41.6]	2.6 (− 1.2 to 6.8)	0.155	0.446
biceps femoris	32.1 [27.1–37.8]	28.5 [19.7–34.8]	4.3 (− 0.4 to 9.6)	0.063	0.373
gluteus maximus	16.4 [8.5–25.9]	19.4 [3.2–26.6]	0.7 (− 6.7 to 8.3)	0.856	0.895
gluteus medius	28.5 [22.8–34.8]	29.3 [20.8–34.2]	0.5 (− 4.7 to 5.8)	0.856	0.895
gluteus minimus	28.1 [18.7–34.2]	23.7 [12.2–33.1]	3.7 (− 3.2 to 11.3)	0.345	0.603
gracilis	38.6 [31.5–41.9]	38.8 [31.5–43.9]	0.0 (− 4.7 to 4.2)	1.000	1.000
iliacus	51.1 [48.6–54.2]	48.5 [47.1–51.7]	1.9 (− 0.8 to 4.3)	0.124	0.446
obturator externus	29.2 [22.6–34.6]	22.4 [15.7–32.1]	5.0 (− 0.8 to 11.1)	0.081	0.373
obturator internus	37.9 [34.4–41.8]	38.0 [28.5–41.7]	1.4 (− 1.6 to 5.8)	0.372	0.603
pectineus	43.8 [38.3–47.4]	41.4 [32.9–45.4]	2.3 (− 1.6 to 6.8)	0.295	0.565
piriformis	34.4 [31.1–38.3]	34.8 [29.5–38.6]	0.3 (− 2.9 to 3.8)	0.846	0.895
psoas major	40.4 [35.3–43.7]	38.5 [32.1–41.2]	2.2 (− 1.7 to 6.4)	0.229	0.527
rectus femoris	47.9 [44.8–50.0]	47.0 [42.8–48.8]	1.2 (− 1.4 to 3.6)	0.393	0.603
sartorius	36.9 [30.4–40.8]	34.3 [28.6–39.2]	2.0 (− 2.2 to 6.7)	0.277	0.565
semimembranosus	29.1 [20.4–38.0]	27.4 [15.2–32.0]	4.7 (− 2.9 to 11.7)	0.229	0.527
semitendinosus	36.8 [31.0–40.1]	36.8 [29.9–40.1]	0.5 (− 3.8 to 4.6)	0.796	0.895
tensor fasciae latae	29.3 [20.9–36.8]	12.7 [–3.1–24.6]	16.4 (7.1 to 25.4)	0.001	0.023
vastus lateralis intermedius	37.7 [29.4–42.7]	34.4 [21.8–40.9]	4.0 (− 1.4 to 9.0)	0.151	0.446
vastus medialis	40.0 [35.2–46.2]	35.4 [27.6–41.6]	5.7 (0.7 to 10.2)	0.022	0.253
anterior compartment	44.4 [40.1–50.3]	41.5 [34.2–46.9]	4.4 (− 0.4 to 10.0)	0.072	0.373
lateral compartment	43.9 [40.2–49.0]	43.4 [35.3–49.8]	0.8 (− 4.1 to 5.9)	0.766	0.895
deep posterior compartment	34.8 [31.1–40.1]	35.3 [24.0–41.1]	1.7 (− 3.0 to 7.4)	0.469	0.635
superficial posterior compartment	34.6 [27.1–38.4]	30.6 [24.4–40.4]	2.0 (− 3.8 to 7.3)	0.457	0.635

Mean HU values were compared between groups. After false discovery rate correction, significantly lower HU values were observed only in the tensor fasciae latae in the valgus group. The vastus medialis showed a lower HU value before correction, but this difference did not remain significant after false discovery rate correction. Lower HU indicates greater fatty degeneration. Values are presented as median [interquartile range]. Between-group differences are presented as Hodges–Lehmann median differences with 95% confidence intervals (CIs). Mann–Whitney U test was used for between-group comparisons. p values are reported as unadjusted values, and q values represent false discovery rate–adjusted p values calculated using the Benjamini–Hochberg procedure. Statistical significance was determined based on q < 0.05. IQR, interquartile range

Fat percentage analysis using Materialise Mimics revealed no significant difference in the vastus medialis; however, the TFL demonstrated significantly greater fat infiltration in the valgus group (Table 4). 

**Table 4:** Intramuscular Fat Percentage of the Vastus Medialis and Tensor Fasciae Latae

	Varus (fat content %)	Valgus (fat content %)	*p*
Tensor fasciae latae	6.5 ± 4.6	17.0 ± 10.4	< 0.001
Vastus medialis	2.9 ± 2.0	3.4 ± 1.8	0.250

Fat volume and fat percentage derived from Mimics segmentation are shown. Although no significant group difference was found in the vastus medialis, the tensor fasciae latae demonstrated significantly higher fat infiltration in the valgus group.

Fat percentage = (fat volume/muscle volume) × 100.

Mann–Whitney *U* test; significance *p* < 0.05.

**Fig. 4:**
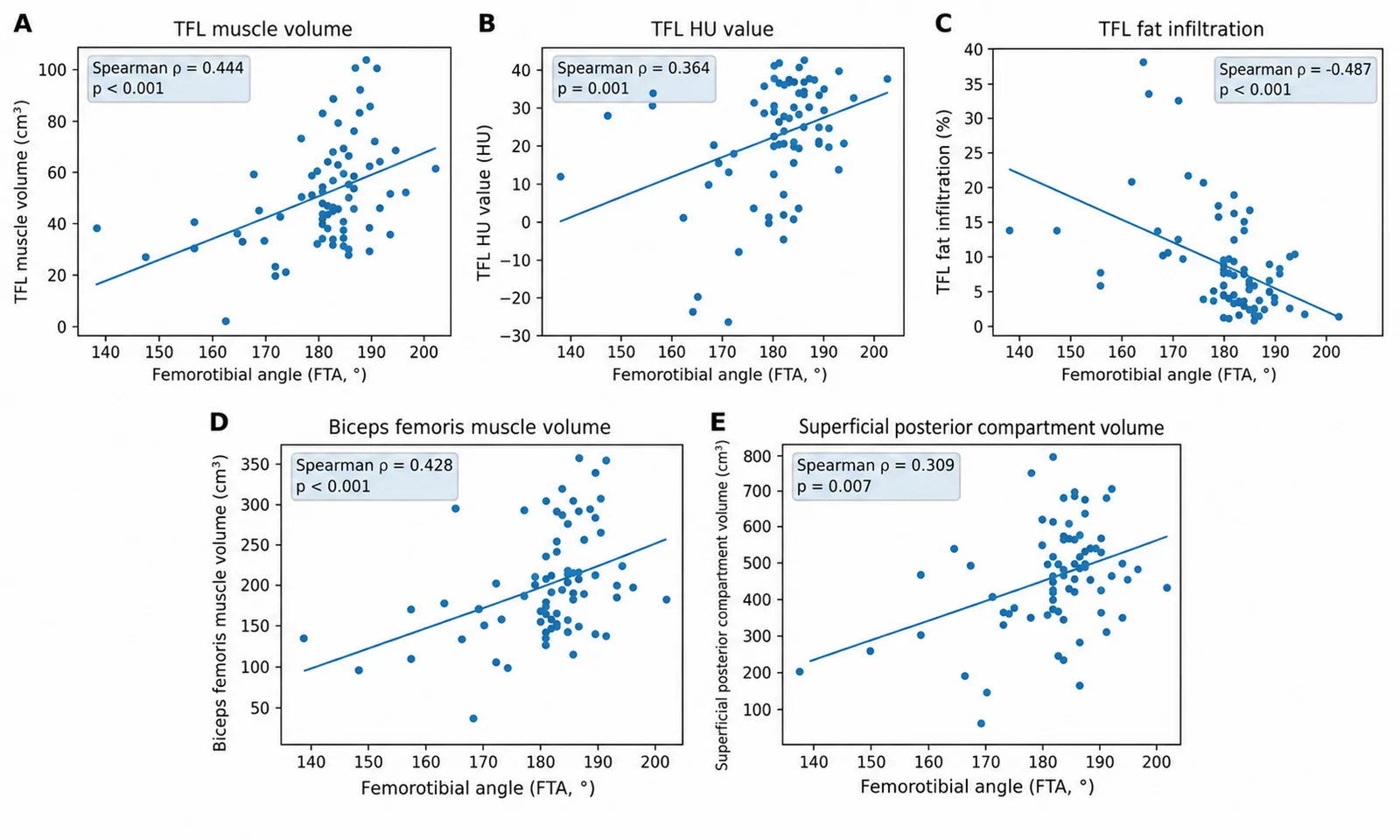
Scatter plots showing the relationships between femorotibial angle (FTA) and representative muscle parameters. **A** FTA and tensor fasciae latae (TFL) muscle volume. **B** FTA and TFL Hounsfield unit (HU) value.** C** FTA and TFL fat infiltration. **D** FTA and biceps femoris muscle volume. **E** FTA and superficial posterior compartment volume. Lower FTA values indicate greater valgus alignment. Spearman’s rank correlation analysis demonstrated that lower FTA values were significantly associated with lower TFL muscle volume, lower TFL HU values, greater TFL fat infiltration, lower biceps femoris muscle volume, and lower superficial posterior compartment volume

To further evaluate the relationship between lower-limb alignment and muscle degeneration, additional Spearman’s rank correlation analyses were performed using FTA as a continuous variable. Lower FTA values, indicating greater valgus alignment, were significantly associated with lower TFL muscle volume (ρ = 0.444, *p* < 0.001), lower TFL HU values (ρ = 0.364, *p* = 0.001), and greater TFL fat infiltration (ρ = -0.487, *p* < 0.001). Lower FTA values were also associated with lower biceps femoris muscle volume (ρ = 0.428, *p* < 0.001) and lower superficial posterior compartment volume (ρ = 0.309, *p* = 0.007) (Fig. 4A–E).

## Discussion

This study found that, compared with varus knee OA, valgus knee OA was associated with reduced muscle volume in the biceps femoris, TFL, and superficial posterior compartment, as well as lower HU values and greater fat infiltration of the TFL. These findings suggest that valgus knee OA may be characterized by both quantitative and qualitative deterioration of the lateral and posterior musculature rather than compensatory strengthening of the medial musculature. Recent research has emphasized the clinical importance of musculoskeletal alterations in knee OA in relation to joint function, injury prevention, and rehabilitation [3]. Contrary to our initial hypothesis, valgus knee OA was not associated with compensatory strengthening or preserved muscle quality of the vastus medialis. Although the vastus medialis had a lower HU value in the valgus group before correction for multiple comparisons, this difference did not remain statistically significant after false discovery rate correction, and Mimics-based fat quantification showed no significant difference in fat percentage. Instead, the most consistent findings were observed in the TFL, which demonstrated reduced muscle volume, lower HU values, and greater fat infiltration in valgus knees. These findings suggest that valgus knee OA may not be explained by simple compensatory hypertrophy of the medial musculature, but rather by deterioration of the lateral and posterior dynamic stabilizers. Since these muscles may contribute to frontal-plane knee control, gait stability, and functional activities [6, 10, 16], their deterioration could be relevant to knee functional status. However, knee functional scores, muscle strength, and gait parameters were not directly assessed in the present study; therefore, future studies should investigate the relationship between alignment-specific muscle degeneration and clinical functional outcomes. Using AI-based segmentation and 3D reconstruction, we assessed whole-lower-limb muscle morphology and fatty degeneration in end-stage OA and clarified the characteristic differences between varus and valgus knees.

Several automated muscle assessment tools have recently been developed. For example, MuscleMap is an open-source, community-supported initiative for whole-body quantitative MRI of muscle [17]. In contrast, the Bayesian U-Net-based pipeline used in the present study was developed specifically for clinical CT images and facilitates 3D segmentation and quantitative assessment of lower-limb muscles and compartments in patients undergoing TKA. A major practical advantage of this approach is that it can utilize CT data routinely acquired for preoperative surgical planning and does not require additional MRI acquisition. Moreover, CT-based analyses enable simultaneous assessment of muscle volume and HU-based muscle quality in a perioperative workflow. However, direct comparisons between CT-based and MRI-based automated muscle assessment tools were beyond the scope of the present study. Future studies should compare the accuracy, generalizability, and clinical utility of these approaches across different imaging modalities and patient populations.

Consistent with previous reports indicating decreased quadriceps strength and hamstring weakness in knee OA [18–20], our results revealed decreased muscle volume in the valgus group, particularly in the biceps femoris, TFL, and superficial posterior compartment. Only one previous study compared muscle volumes between varus and valgus knees, and it assessed only the gastrocnemius muscle [21]. The present whole-limb evaluation provides new insights by identifying specific patterns of volume loss and disproportionate fatty degeneration associated with valgus deformities. Previous studies have relied on single-slice CT or MRI measurements [3, 7], which are limited by slice-selection bias and cannot accurately reflect whole-muscle morphology. In contrast, our 3D analysis minimized this bias and enabled a more comprehensive and reliable assessment of muscle volume.

Our findings may suggest weakening of the posterior and lateral supporting structures of the knee in patients with valgus OA. In contrast, no marked atrophy of these muscle groups was observed in varus knees, in which the muscle volumes were relatively preserved. This whole-limb analysis therefore provides additional insight into alignment-specific muscle involvement in knee OA.

Hip abductor weakness is frequently reported in knee OA [22, 23]. Reduced TFL volume is consistent with hip abductor weakness. In valgus knees, pain localized near the lateral compartment (close to the insertion sites of the biceps femoris and iliotibial band) may cause patients to avoid movements that require strong activation of these structures. Furthermore, lateral compartment involvement may be associated with worse knee pain [24], which could contribute to reduced physical activity [25] and disuse-related muscle degeneration in the superficial posterior compartment. Similarly, the gastrocnemius–soleus complex may experience reduced loading because of pain and altered gait patterns in valgus OA.

The significantly greater fat infiltration in the TFL in the valgus group suggests qualitative deterioration of muscle function because fatty infiltration reflects reduced contractile capacity [15]. As the TFL stabilizes the lateral knee through the iliotibial band [16], its functional decline may contribute to lateral knee instability or altered frontal-plane control. Importantly, fat infiltration should be interpreted as a qualitative muscle alteration distinct from muscle volume loss. While reduced muscle volume may reflect atrophy or decreased mechanical demand, increased intramuscular fat suggests replacement or infiltration of contractile tissue by non-contractile components, which may impair force generation even when muscle size is partially preserved [26]. In the present study, the TFL demonstrated both reduced volume and increased fat infiltration in valgus knees, indicating that valgus alignment may be associated with combined quantitative and qualitative deterioration of this lateral stabilizing muscle. This finding suggests that evaluation of fat infiltration provides additional information beyond muscle volume alone when assessing alignment-related muscular changes in knee OA [10, 27]. Since the TFL contributes to lateral knee stability through the iliotibial band [16], such qualitative deterioration may be particularly relevant in valgus alignment, where lateral compartment loading and frontal-plane knee control are clinically important [28].

CT attenuation is influenced not only by fat content but also by water content, edema, and fibrotic tissue [29]. HU values are also affected by scanner settings and reconstruction kernels, which may introduce further variability. In contrast, Mimics separates fat and muscle voxels based on HU thresholds, providing a more direct measure of fat infiltration. Therefore, we considered the Mimics-based fat percentage as the primary metric for assessing intramuscular fat infiltration in the present study. Additionally, both the adjusted HU values and Mimics-based fat quantification revealed a consistent increase in the TFL fat content, and no inconsistency was observed between the two approaches.

The present findings may provide a clinical basis for considering alignment-specific rehabilitation strategies in patients with knee OA [3, 10]. Although rehabilitation for knee OA often predominantly emphasizes quadriceps strengthening, our results suggest that valgus knee OA may be associated with deterioration of the lateral and posterior muscle groups, particularly the TFL, biceps femoris, and superficial posterior compartment. These findings imply that assessment and treatment of muscles involved in lateral knee stability and posterior chain function may be relevant in the management of valgus knee OA. For example, rehabilitation programs may need to incorporate hip abductor and lateral stabilizer training, posterior chain strengthening, gait training, and balance exercises, in addition to conventional quadriceps-focused interventions. 3D assessment of muscle volume and fat infiltration may help identify patient-specific muscular deficits and support individualized preoperative and postoperative rehabilitation planning for patients undergoing TKA [10]. Since the TFL contributes to lateral knee stability through the iliotibial band, targeted assessment of this muscle may be particularly relevant in valgus knee OA [16].

### Limitations

This study has several limitations. First, only patients with end-stage OA scheduled for TKA were included, which limits the generalizability of these findings to earlier OA stages. Second, the valgus group contained fewer patients than the varus group, reflecting the lower prevalence of valgus knee OA in our surgical population. This imbalance may have reduced the statistical power to detect modest between-group differences, particularly after false discovery rate correction. Consequently, some clinically relevant trends may not have reached statistical significance. Future multicenter studies with larger and more balanced cohorts are needed to confirm these findings. Third, although we performed a supplementary analysis using body-weight-normalized muscle volume, residual confounding related to body composition may persist. Body weight does not distinguish between lean mass and fat mass, and ratio-based normalization may introduce statistical bias. Therefore, future studies should consider more sophisticated normalization methods, such as adjustment for lean body mass or skeletal size. Fourth, the AI-based segmentation model was originally validated using hip-to-knee CT images, and quantitative validation for the lower-leg muscle compartments was not performed in the present study. Although all segmentation results were visually inspected by an orthopedic surgeon and no obvious segmentation errors warranting correction or exclusion were identified, visual quality control cannot substitute for quantitative validation against manual segmentation. Therefore, the results for the lower-leg compartments should be interpreted with caution, and future studies should validate the model quantitatively for lower-leg muscle segmentation. Fifth, the cross-sectional design precludes causal inference; whether muscle atrophy precedes deformity or results from it remains unclear. Although muscle assessment was performed using CT, repeated imaging was limited by the radiation exposure. Therefore, future research should consider non-ionizing modalities, such as MRI or ultrasonography, for longitudinal evaluation. Sixth, while we focused on morphological parameters, such as muscle volume and fatty degeneration, we did not assess muscle function (e.g., strength testing, gait analysis, or neuromuscular control). Accordingly, the functional interpretations of our findings should be interpreted cautiously. Seventh, although the false discovery rate control was applied, the results should be interpreted in the context of the multiple testing conducted, and thus warrant confirmation in independent cohorts.

## Conclusions

CT-based 3D analysis using AI demonstrated that valgus knees exhibit characteristic reduction in the biceps femoris, TFL, and superficial posterior compartment, along with increased fatty infiltration of the TFL. Varus and valgus knees show distinct morphological and qualitative muscle characteristics that may inform individualized prevention, treatment, and rehabilitation strategies for knee OA.

## Supplementary Information

Additional file 1: Supplementary Table 1 contains supporting data referenced in the main manuscript, provided to complement the primary results. Supplementary Table 1. Comparison of Body-Weight-Normalized Muscle Volume Between Varus and Valgus Knees.

## Data Availability

The datasets generated and analyzed in the current study are available from the corresponding author upon reasonable request.

## Abbreviations

OA: Osteoarthritis
TKA: Total knee arthroplasty
FTA: Femorotibial angle
BMI: Body mass index
TFL: Tensor fasciae latae
CT: Computed tomography
MRI: Magnetic resonance imaging
3D: Three-dimensional
AI: Artificial intelligence
HU: Hounsfield units
