# Correction: Three-dimensional artificial intelligence-based computed tomography analysis of lower limb muscle volume and fatty degeneration in varus and valgus knee osteoarthritis: a single-center retrospective study

**DOI:** 10.1186/s12891-026-10492-x

**Published:** 2026-10-24

**Authors:** Yoshinobu Hyakuda, Takuma Miyamoto, Munehiro Ogawa, Akihito Kawai, Hiroto Nishimura, Ryota Hara, Yusuke Inagaki, Keisuke Uemura, Masaki Takao, Kohei Kono, Mazen Soufi, Yoshito Otake, Kenji Kawamura

**Affiliations:** 1 Department of Orthopaedic Surgery, Nara Medical University, 840 Shijocho, Kashihara, Nara 634-8521 Japan; 2 Department of Sports Medicine, Nara Medical University, Kashihara, Japan; 3 The Center for Rheumatic Diseases, Nara Medical University, Kashihara, Japan; 4 Department of Rehabilitation Medicine, Nara Medical University, Kashihara, Japan; 5 Department of Orthopaedic Medical Engineering, Osaka University Graduate School of Medicine, Suita, Japan; 6 Department of Orthopaedic Surgery, Ehime University Graduate School of Medicine, Toon, Japan; 7 Program of Information and Communication Engineering, Faculty of Engineering, University of Miyazaki, Miyazaki, Japan; 8 Division of Information Science, Nara Institute of Science and Technology, Ikoma, Japan

**Correction: BMC Musculoskelet Disord 27, 677 (2026)**

**https://doi.org/10.1186/s12891-026-10034-5**

Following publication of the original article [1], the authors identified an error in Figure 2. “surgical history on the affected lower extremity (n = 16)” was missing.

The incorrect and the correct figure is given below.

The incorrect figure 2

**Figure:**
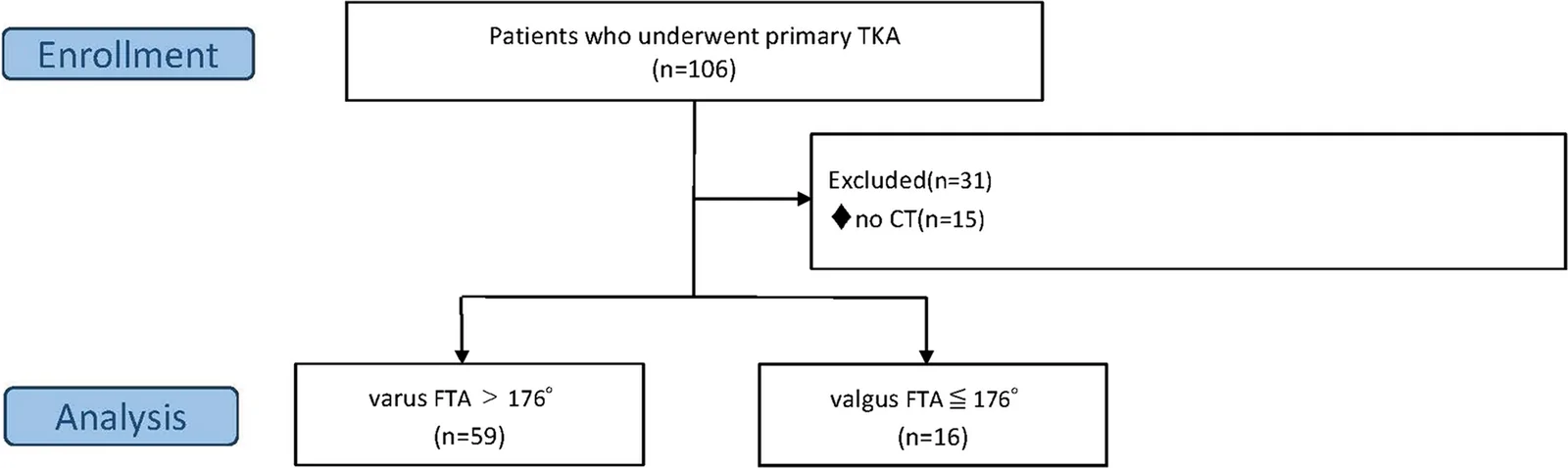

The correct figure 2

**Figure:**
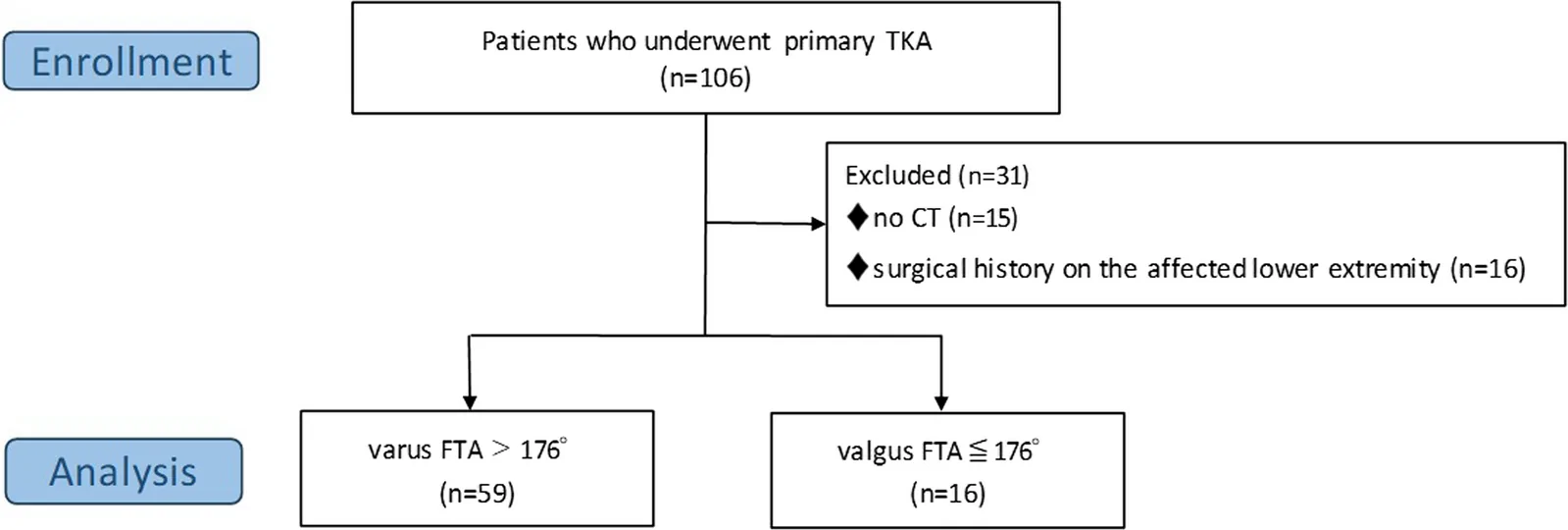

The original article has been updated.
