# Supplementary material for: Three-dimensional artificial intelligence-based computed tomography analysis of lower limb muscle volume and fatty degeneration in varus and valgus knee osteoarthritis: a single-center retrospective study
Source: BMC Musculoskelet Disord. 2026 Jun 5;27:677. doi: 10.1186/s12891-026-10034-5 (PMC13459205; doi:10.1186/s12891-026-10034-5)
Supplement: Supplementary file 1 [file 12891_2026_10034_MOESM1_ESM.docx]

**Supplementary Table 1. Comparison of Body-Weight-Normalized Muscle Volume Between Varus and Valgus Knees**

| Muscle/Compartment | Varus [cm³/kg] (median [IQR]) | Valgus [cm³/kg] (median [IQR]) | Between-group difference (median difference [95% CI]) | p | q |
| --- | --- | --- | --- | --- | --- |
| adductor | 9.76 [8.42–10.70] | 8.60 [7.26–9.06] | 1.35 (0.37 to 2.05) | 0.010 | 0.047 |
| biceps femoris | 3.20 [2.81–3.93] | 2.58 [2.26–2.80] | 0.73 (0.38 to 1.16) | <0.001 | 0.003 |
| gluteus maximus | 10.30 [9.43–11.51] | 10.35 [9.06–11.01] | 0.32 (−0.39 to 1.33) | 0.497 | 0.612 |
| gluteus medius | 4.07 [3.60–4.56] | 4.10 [3.77–4.34] | −0.04 (−0.39 to 0.31) | 0.861 | 0.901 |
| gluteus minimus | 0.87 [0.76–0.97] | 0.87 [0.69–0.95] | 0.03 (−0.06 to 0.14) | 0.473 | 0.612 |
| gracilis | 0.82 [0.68–0.99] | 0.80 [0.66–0.96] | 0.01 (−0.15 to 0.12) | 0.912 | 0.912 |
| iliacus | 1.48 [1.29–1.68] | 1.45 [1.21–1.70] | 0.05 (−0.14 to 0.25) | 0.609 | 0.701 |
| obturator externus | 0.58 [0.52–0.66] | 0.53 [0.49–0.61] | 0.05 (0.00 to 0.10) | 0.075 | 0.193 |
| obturator internus | 0.59 [0.49–0.66] | 0.59 [0.51–0.64] | 0.00 (−0.05 to 0.07) | 0.851 | 0.901 |
| pectineus | 0.49 [0.44–0.57] | 0.47 [0.40–0.52] | 0.04 (−0.02 to 0.09) | 0.172 | 0.283 |
| piriformis | 0.31 [0.26–0.37] | 0.38 [0.32–0.42] | −0.05 (−0.10 to 0.00) | 0.056 | 0.162 |
| psoas major | 1.22 [1.02–1.44] | 1.08 [0.87–1.23] | 0.14 (−0.07 to 0.35) | 0.164 | 0.283 |
| rectus femoris | 1.86 [1.64–2.13] | 1.62 [1.50–1.77] | 0.24 (0.07 to 0.39) | 0.009 | 0.047 |
| sartorius | 1.51 [1.33–1.77] | 1.24 [1.15–1.56] | 0.21 (0.00 to 0.35) | 0.046 | 0.150 |
| semimembranosus | 2.26 [1.94–2.68] | 2.19 [1.81–2.52] | 0.15 (−0.15 to 0.50) | 0.280 | 0.430 |
| semitendinosus | 1.89 [1.62–2.11] | 1.53 [1.43–1.92] | 0.26 (0.01 to 0.45) | 0.033 | 0.128 |
| tensor fasciae latae | 0.81 [0.67–1.01] | 0.61 [0.46–0.68] | 0.26 (0.13 to 0.39) | <0.001 | 0.003 |
| vastus lateralis intermedius | 8.21 [7.06–9.24] | 7.35 [6.63–7.97] | 0.72 (−0.21 to 1.58) | 0.146 | 0.279 |
| vastus medialis | 3.54 [2.92–4.07] | 3.26 [2.99–3.37] | 0.31 (−0.03 to 0.70) | 0.129 | 0.279 |
| anterior compartment | 2.45 [1.79–2.86] | 2.25 [1.93–2.55] | 0.16 (−0.22 to 0.49) | 0.505 | 0.612 |
| lateral compartment | 1.12 [0.94–1.34] | 0.97 [0.70–1.26] | 0.16 (−0.06 to 0.39) | 0.142 | 0.279 |
| deep posterior compartment | 3.00 [2.47–3.53] | 3.00 [2.23–3.34] | 0.20 (−0.22 to 0.70) | 0.376 | 0.540 |
| superficial posterior compartment | 8.33 [6.30–9.25] | 5.82 [4.01–7.50] | 2.10 (0.59 to 3.80) | 0.005 | 0.041 |
| Body-weight-normalized muscle volume was calculated as muscle volume divided by body weight (cm³/kg). Values are presented as median [interquartile range]. Between-group differences are presented as Hodges–Lehmann median differences with 95% confidence intervals (CIs). Mann–Whitney U test was used for between-group comparisons. p values are reported as unadjusted values, and q values represent false discovery rate–adjusted p values calculated using the Benjamini–Hochberg procedure. Statistical significance was determined based on q < 0.05. IQR, interquartile range | | | | | |
